# Correlation of IL-6 and JAK2/STAT3 signaling pathway with prognosis of nasopharyngeal carcinoma patients

**DOI:** 10.18632/aging.203186

**Published:** 2021-06-22

**Authors:** Mengqi Zhuang, Xiaotong Ding, Wenli Song, Huimin Chen, Hui Guan, Yang Yu, Zicheng Zhang, Xinzhe Dong

**Affiliations:** 1 Department of Oncology, The Fourth People’s Hospital of Jinan, Jinan 250031, PR China; 2 Department of Oncology, Jinan Fuda Cancer Hospital, Jinan 250033, PR China; 3 Department of Clinical Laboratory, The Fourth People’s Hospital of Jinan, Jinan 250031, PR China; 4 Department of Radiation Neurology, The Fourth People’s Hospital of Jinan, Jinan 250031, PR China; 5 Department of Radiation Oncology, The Fourth People’s Hospital of Jinan, Jinan 250031, PR China; 6 School of Graduate Studies, Shandong First Medical University and Shandong Academy of Medical Sciences, Jinan 271099, PR China; 7 Department of Radiation Oncology, Shenzhen Traditional Chinese Medicine Hospital, The Fourth Clinical Medical of Guangzhou University of Chinese Medicine, Shenzhen 518033, PR China; 8 Department of Radiation Oncology, Qilu Hospital of Shandong University, Jinan 250012, PR China

**Keywords:** IL-6, JAK2/STAT3 signaling pathway, clinicopathological features, prognosis, nasopharyngeal carcinoma

## Abstract

IL-6 is reported to be the main upstream activator, instead of the downstream target of JAK2/STAT3. This study is intended to explore the correlation of IL-6 and JAK2/STAT3 signaling pathway with clinicopathological features and prognosis in nasopharyngeal carcinoma (NPC). First, NPC tissues and normal nasopharyngeal epithelial tissues were obtained from 117 NPC patients. Next, we detected expression levels of IL-6 in serum and those of STAT3, p-STAT3, JAK2, p-JAK2 and CyclinD1 in tissues. A follow-up was conducted in all the patients and the survival was analyzed. To verify the correlation of IL-6 and JAK2/STAT3 pathway, CNE-1 and SUNE1 NPC cells were interpreted with IL-6 and JAK2/STAT3 signaling pathway inhibitor AG490 to detect cell viability, migration and invasion. We observed thatIL-6 increased in serum of NPC patients. The expressions of IL-6, STAT3, p-STAT3, JAK2, p-JAK2 and CyclinD1 in NPC tissues were higher and correlated with TNM stage and lymph node metastasis (LNM). Survival rates were reduced in patients with positive expressions of IL-6, STAT3, p-STAT3, JAK2, p-JAK2 and CyclinD1. LNM and positive expressions of IL-6 and p-STAT3 were risk factors for poor prognosis of NPC. Besides, recombinant human IL-6 promoted cell proliferation, invasion and migration while AG490 inhibited cell proliferation, invasion and migration in CNE-1 and SUNE1 NPC cells. The results demonstrated that increased IL-6 expression and the activated JAK2/STAT3 signaling pathway had effects on prognosis and reduced the survival time in NPC patients, which provide a potential target for the treatment of NPC.

## INTRODUCTION

Nasopharyngeal carcinoma (NPC) is a common malignant tumor in the head and neck originating from nasopharyngeal epithelial cells with remarkable variations in ethnic [1] and geographical distribution [2–4]. The highest occurrence rate of NPC is discovered in Southern China, where its incidence is approximately more than 25 cases per 100,000 individuals [5]. Because of secluded anatomical sites and nonspecific symptoms in the early stage, 80% to 90% NPC patients are not diagnosed until the late advanced stage [6]. Currently, radiotherapy has served as the primary treatment of NPC combined with chemotherapy, so the overall survival rate of NPC patients has improved [7]. However, the 5-year survival rate is not ideal in spite of great improvements in radiotherapy and chemotherapy treatments [8]. The predominant cause of therapeutic failure for NPC is metastasis to cervical lymph nodes, thus resulting in a reduction in the overall survival rate [9]. Nowadays, targeted therapy has been a hot spot in cancer treatment. EGFR-targeted therapies [10–12] and VEGF-targeted therapies [13–14] may be an effective strategy for NPC, but the results are inconsistent. So, it is extremely crucial to further understand the molecular mechanisms in NPC tumorigenesis and select effective diagnostic biomarkers and new therapeutic targets in order to improve the survival rate and prognosis of NPC patients [15].

Inflammatory mediators, including TNF-α, IL-6, TGF-β, and IL-10, have been widely incriminated in chronic inflammation and the progression of cancers [16–17]. The pathological feature of NPC tumor microenvironment (TME) is that it releases a large amount of inflammatory messengers such as cytokines (TNF-α, IL-6), which causes immune cell infiltration and promotes tumorigenesis [18]. IL-6 is a pleiotropic cytokine that regulates cell proliferation and inhibits apoptosis and has been proven to overexpress in many types of tumors, such as colon, liver, breast, brain tumor and NPC; furthermore, IL-6 activates multiple pro-proliferation and anti-survival proteins to stimulate growth of tumor cells [19–21]. By binding to its cognate receptor on the cell membrane (IL-6R), IL-6 transduces their signals via membrane-bound glycoprotein 130 (gp130) mostly to indicate transducers and activators of transcription 3 (STAT3) [22, 23]. After stimulation by IL-6, the gp130 forms a dimmer and activates the Janus-activated kinase (JAK1, JAK2 and Tyk2) signal transducer and activator of transcription (JAK/STAT) signaling pathway, especially STAT3 [24]. The JAK/STAT signaling pathway is the main pathway of IL-6, which is constitutively activated by IL-6 and frequently observed in a variety of human cancers, including NPC [25–27]. IL-6 promotes NPC migration of bystander tumor cells by IL-6R/JAK/STAT3 pathway [28]. JAK2/STAT3 signaling pathway plays a critical role in the occurrence and development of tumor cells [29]. STAT3 will be activated by JAK2 when cells receive the pro-proliferative stimulation, thus leading to modulate cell survival and proliferation through its down-stream targets [30]. In previous researches, abnormal JAK2/STAT3 signaling has been observed in lung, gastric, and prostate cancers [31–33]. Therefore, JAK2/STAT3 may be used as a potential target for cancer therapy. The aim of this e study was analyzing the clinicopathological features of NPC and correlation between NPC and prognosis with IL-6 and JAK2/STAT3 signaling pathway.

## MATERIALS AND METHODS

### Ethical statement

The study was approved by the Ethics Committee of Qilu Hospital, Shandong University. All the patients and their families have signed written informs of consent.

### Sample collection

Between January 2007 and December 2009, a total of 117 NPC patients (age range from 18 to 73 years old, with a median age of 46 years old) were selected from Qilu Hospital. Pathological grades of tissue specimens were assessed in accordance with the World Health Organization (WHO) pathological diagnosis criteria [34]. Tumor node metastasis (TNM) staging was assessed in accordance with the American Joint Committee on Cancer (AJCC) 8th edition [35]. There were 9 cases with squamous carcinoma, 93 cases with poorly differentiated squamous carcinoma, 9 cases with undifferentiated carcinoma and 6 cases with adenosquamous carcinoma. There were 51 cases without lymph node metastasis (LNM) and 66 cases with LNM. There were 10 cases in TNM stage I, 26 cases in stage II, 39 cases in stage III, and 42 cases in stage IV. NPC tissues and normal nasopharyngeal epithelial tissues were collected during biopsy, and then part of them were stored in liquid nitrogen and fixed with 10% formaldehyde. Serum samples from each patient were taken as the NPC group, and serum samples of 112 healthy subjects were collected as the control group. All the samples were confirmed by pathological examinations, and none of the included NPC patients received radiotherapy or chemotherapy before operation.

### Enzyme-linked immunosorbent assay (ELISA)

The concentration of IL-6 in serum was measured, respectively using ELISA Kit (ZhongShan JohnKing Pharmaceutical Co., Zhongshan, China) according to the manufacturer’s instructions. An enzyme-labeling plate was placed at room temperature for 30 min, and 100 μl standard samples were then added into 6 wells. Each well was supplemented with 100 μl serum samples. Then each well was added with 50 μl enzyme-labeling solution and placed at room temperature for 90 min. Subsequently, the plate was washed 5 times (10–20 s every time). Each well was added with 50 μl A solution and 50 μl B solution as substrates and incubated for 15 min in the dark. Then 50 μl stop solution was added into each well to terminate the reaction. The optical density (OD_450 nm_) value of each well was measured by the microplate reader (Thermo Fisher Scientific, Carlsbad, CA, USA)

### Immunohistochemistry (IHC)

The NPC tissues and normal nasopharyngeal epithelial tissues were collected, fixed with 10% formaldehyde, paraffin-embedded and cut into sections (4 μm). The sections were incubated at 65°C for 30 min, deparaffinized, hydrated and washed 3 times with double distilled water. Sections were soaked in 3% H_2_O_2_ for 15 min and washed 3 times with phosphate-buffered saline (PBS, 0.01 mol/L). Then sections were soaked in citrate buffer, boiled for 15 min and fully cooled. The sections were washed 3 times again with PBS and incubated with rabbit anti-human primary antibodies IL-6 (Cat# ab6672, 1:500, Abcam, Cambridge, MA, USA), JAK (Cat# ab39636, 1:100, Abcam, Cambridge, MA, USA), p-JAK (Cat# ab32101, 1:100, Abcam, Cambridge, MA, USA), STAT3 (Cat# 119352, 1:600, Abcam, Cambridge, MA, USA), p-STAT3 (Cat# ab76315, 1:100, Abcam, Cambridge, MA, USA) and CyclinD1 (Cat# ab134175, 1:100, Abcam, Cambridge, MA, USA) overnight in the refrigerator at 4°C. After being washed with PBS 3 times, the sections were incubated with HRP-conjugated secondary antibodies (1:5,000; cat. no.S A00004-10, Proteintech Group, Inc.) for 20 min at 37°C and then incubated with SP solution at 37°C for 20 min. Then, the sections were stained with diaminobenzidine (DAB) (Maxim Biotechnology Company, Fuzhou, Fujian, China) and re-stained with hematoxylin. After dehydration and mounting, the sections were viewed using a microscope Olympus BX51 (Olympus Optical Co., Ltd, Tokyo, Japan). Staining cells usually presented brownish yellow granules. The staining intensity of cells was scored as follows: uncolored or not obvious = 0 point, light yellow = 1 point, brownish yellow = 2 points and brown = 3 points. The percentage of positive cells was scored as < 5% = 0 point, 5%–25% = 1 point, 26%–50% = 2 points and > 50% = 3 points. Final scores were calculated by the addition of two scores. The score was 0–1 point as negative (–), 2–3 points as weakly positive (+) and 4–6 points as positive (++). In this study, both “–” and “+” were regarded as negative, “++” as positive.

### Reverse transcription quantitative polymerase chain reaction (RT-qPCR)

The RNA from the NPC tissues and normal nasopharyngeal epithelial tissues was extracted by RNeasy mini Kit (QIAGEN, GmbH, Germany). RNA was dissolved by ultra-pure water treated with diethylpyrocarbonate, and ND-1000 ultraviolet/visible spectrophotometer (Nanodrop, Waltham, MA, USA) was used to measure the OD value at 260 nm and 280 nm. Besides, the total RNA quality was identified and determined. Then total RNA reverse transcription was performed using PrimeScript RT Kit (RR014A, Takara Biomedical Technology Co., Ltd, Beijing, China). TaqMan Universal PCR kit (Applied Biosystems, CA, USA) was employed for RT-qPCR. The thermocycling conditions were: 95°C for 10 min and 40 cycles of 95°C for 15 s and 60°C for 1 min. ABI 7500 (Applied Biosystems, Inc., CA, USA) was used for RT-qPCR. Three duplicate wells were set for each sample with glyceraldehyde-3-phosphate dehydrogenase (GAPDH) as an internal control. Primer sequences used in the present study are given in Table 1. And the primers were synthesized by Invitrogen (Invitrogen, Shanghai, China). The 2^-ΔΔCt^ method was used to calculate the relative quantification.

**Table 1:** Primer sequences for RT-qPCR.

**Genes**	**Primer sequences**
IL-6	F: 5′-AAATTCGGTACATCCTCGACGG-3′
	R: 5′-GGAAGGTTCAGGTTGTTTTCTGC-3′
STAT3	F: 5′-GTCAGAT -GCCAAATGCG-3′
	R: 5′-TGATGTACCCT -TCGTTCT-3′
JAK2	F: 5′-GGGTGTTCGCGTCGCCACTT-3′
	R: 5′-CAGATCGGGCGAC-CAGAGCGC-3′
CyclinD1	F: 5′-GCTGCGAAGTGGAAACCATC-3′
	R: 5′-CCTCCTTCTGCACACATTTGAA-3′
GAPDH	F: 5′-ACCACAGTCCATGCCATCAC-3′
	R: 5′-TCCACCACCCTGTTGCTGTA-3′

Abbreviations: RT-qPCR: reverse transcription quantitative polymerase chain reaction; IL-6: interleukin-6; STAT3: Signal transducers and activators of transcription 3; JAK2: Janus kinase 2; GAPDH: glyceraldehyde-3-phosphate dehydrogenase.

### Western blot analysis

The NPC tissues and normal tissues were washed with PBS, added with cell lysates containing with protease inhibitors, shaken at 4°C for 5 min and centrifuged at 4°C for 10 min at the rate 37100 × g. The proteins were extracted from the supernatant by using Qproteome Mammalian Protein Prer kit (QIAGEN, GmbH, Germany). Proteins (50 μg) were obtained for sodium dodecyl sulfate-polyacrylamide gel electrophoresis (SDS-PAGE) and then moved to nitrocellulose membranes. Following blocking with skimmed milk and incubated overnight with the following primary antibody IL-6 (Cat# ab9324, 0.4 μg/ml, Abcam, Cambridge, MA, USA), JAK (Cat# ab205223, 1: 500, Abcam, Cambridge, MA, USA), p-JAK (Cat# ab32101, 1: 1000, Abcam, Cambridge, MA, USA), STAT3 (Cat# 119352, 1:600, Abcam, Cambridge, MA, USA), p-STAT3 (Cat# ab76315, 1:2000, Abcam, Cambridge, MA, USA) and CyclinD1 (Cat# ab134175, 1: 10000, Abcam, Cambridge, MA, USA). After washing with tris-buffered saline solution containing Tween 20 (TBST) 4 times (10 min every time), the membranes were incubated with IRDye™ 700DX-labeled IgG (LI-COR Bioscience, NE, USA) antibody (dilution 1:1000, Upstate, NY, USA) at room temperature for 1 h, washed by TBST 4 times and developed with the substrates. The data were analyzed by LabWorks Image Acquisition and Analysis Software (UVP, Inc., Upland, CA, USA) to obtain the relative protein concentration.

### Follow-up

Follow-up was performed by telephone or letter once every 2 months to observe the survival of patients. Follow-up began at the time of pathological diagnosis and ended in December 2015, lasting for 6 years. Among 117 patients, 4 were dropped to follow-up, and the rate of loss to follow-up was 4.27%.

### Cell culture and treatment

NPC CNE-1 cells and SUNE1 cells were obtained from Shanghai Institute of Cell Bank. The CNE-1 cells and SUNE1 cells were inoculated into a RPMI 1640 culture medium with 10% fetal bovine serum (FBS) and 1% penicillin-streptomycin in an incubator with 5% CO_2_ at 37°C. Subculture (1:3) was carried out when cell confluence reached 90%. The cells in logarithmic growth phase were placed on a 6 well-plate (1 × 10^5^ cells/well, 3 ml nutrient fluid each well) and the following experiment was carried out when cell confluence reached 50% to 70%. AG490, which is used to selectively inhibit JAK/Stat-3 activation, inhibits the activation of Stat-3 by selectively blocking JAK2 [36]. AG490 could also inhibit cell proliferation and induce cell apoptosis by blocking the activation of JAK2 mediated by IL-6 [37]. The CNE-1 cells and SUNE1 cells were grouped into five groups: blank group (equal volume of culture medium), NC group (equal density of dimethyl sulfoxide (DMSO) culture medium), IL-6 group (100 ng/mL recombinant human IL-6 culture medium, Peprotech, Rocky Hill, NJ, USA) [38], AG490 group (50 uM JAK2/STAT3 signaling pathway inhibitor AG490, Alexis, Philadelphia, PA, USA; [39], and IL-6 + AG490 group (100 ng/mL recombinant human IL-6 + 50 uM AG490).

### Cell counting kit-8 (CCK-8) assay

The CNE-1 cells and SUNE1 cells were placed on a 96-well plate at density of 2 × 10^3^ cells/well. After cells adhered to the well surface, the culture medium was replaced by 100 ng/mL recombinant human IL-6, 50 uM AG490 and co-cultured medium, respectively. Six duplicated wells were set in each group, and 20 ul CCK-8 solution was added into each well for 2-h incubation at 37°C before examination. The examination was conducted one time each day for 5 days. At last, the OD_450 nm_ wavelength was detected by a microplate reader.

### Scratch test

The CNE-1 and SUNE1 cells (after 24 h reaction) in logarithmic growth phase was placed in 6-well plate (5 × 10^3^ cell each well) in an incubator at 37°C with 5% CO_2_. After the cells were adhered to the monolayer cells, the sterilized 10 μL micropipette was linearly scratched in the 6-well plate, and the D-hanks was used to remove the detached cells. And the cells were added with the serum-free culture solution. There were 3 duplicated wells in each group, and the samples were selected after 0 h and 48 h scratching. Three visual fields (100×) were selected and photographed under a phase contrast microscope. Image pro plus software was used to detect migration distance in each group, and the study was repeated 3 times.

### Transwell assay

After 24 h reaction, the CNE-1 and SUNE1 cells were collected and washed carefully with PBS one time. The cells were suspended in DMEM culture medium (containing 10% FBS). The cell concentration was adjusted to 10^5^ cells/per well, and 100 ul in each well in the apical chamber of Transwell. Culture medium was added into the basolateral chamber at 37°C in a 5% CO_2_ atmosphere for 12 h. The chamber was then taken out, and the above medium was removed. Cells not passing the membrane were wiped out by cotton swabs and then the remaining cells were fixed with 4% polyoxymethylene for 10 min, followed washing with distilled water twice, 5 min a time and dried out by wind. Crystal violet (0.1%) was employed for staining for 15 min, and then miscellaneous liquid was removed, and then the cells was dried by wind and sealed. The slide was viewed and photographed under an inverted optical microscope. Next, 4 visual fields were randomly selected, and cells passing through the microporous membrane were counted and the average value was calculated. The experiment was repeated 3 times independently.

### Statistical analysis

Applying the statistical package for the social sciences (SPSS) version 19.0 (IBM Corp., Armonk, NY, USA) for data analyses. Results were displayed as mean ± standard deviation. The comparisons between two groups were analysed by *t* test. After homogeneity test of variance, multiple groups were compared using one-way analysis of variance (ANOVA). The pairwise comparison of means in multiple groups was performed by least significant difference (LSD)-*t* test. Survival analysis was performed applying Kaplan-Meier method and log-rank test. Cox proportional hazard model was used for multivariate analysis. Values of *p* < 0.05 were regarded as indicate a statistically significant difference.

## RESULTS

### IL-6, STAT3, p-STAT3, JAK2, p-JAK2 and CyclinD1 expressions are associated with the progression and metastasis of NPC

**Figure 1:**
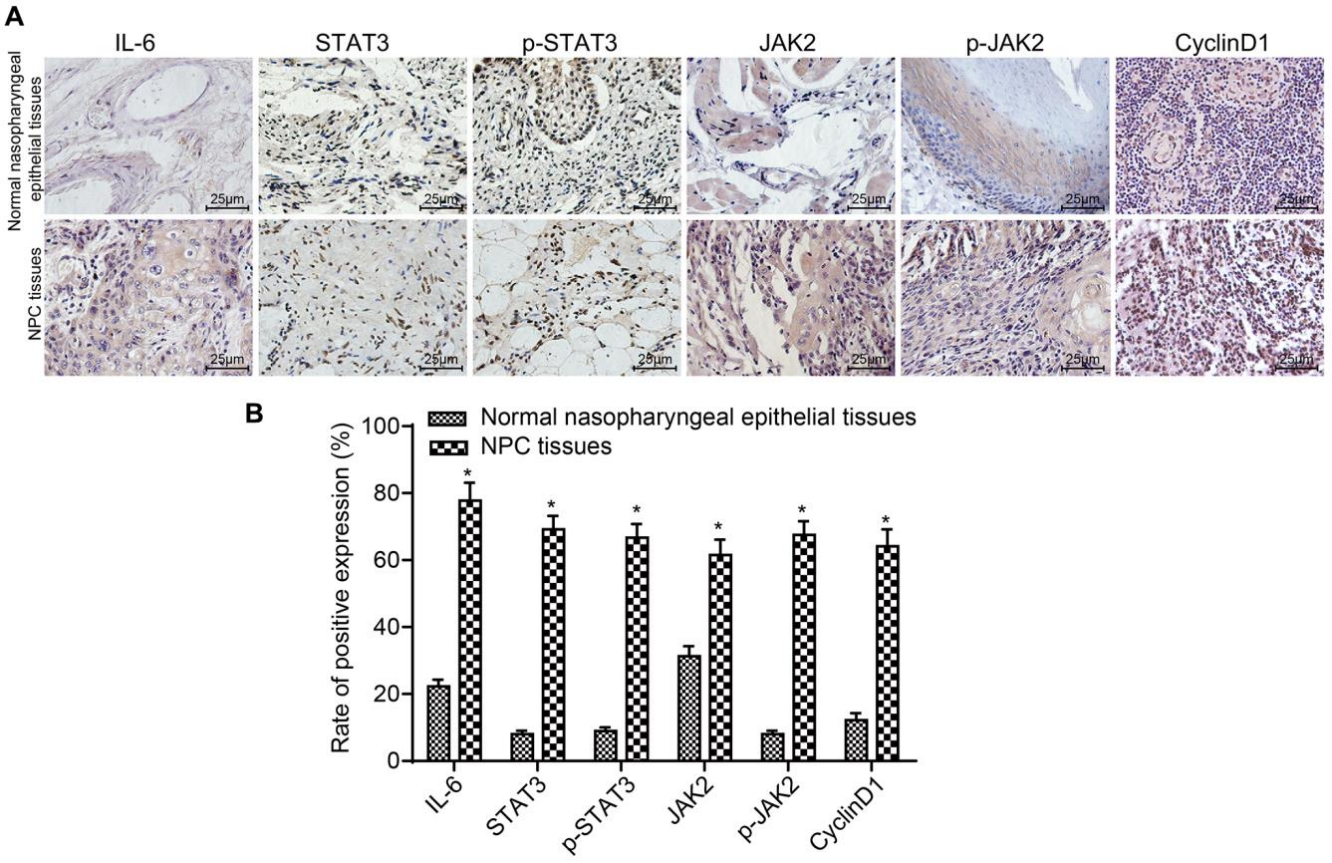
**The positive rates of IL-6, STAT3, p-STAT3, JAK2, p-JAK2 and CyclinD1 were obviously higher in NPC tissues.** (**A**) The results of immunohistochemical staining in NPC tissues and normal nasopharyngeal epithelial tissues (× 400); (**B**) The positive expression rates of IL-6, STAT3, p-STAT3, JAK2, p-JAK2 and CyclinD1 in NPC tissues (*n* = 117) and normal nasopharyngeal epithelial tissues (*n* = 112) (5 sections selected in each result); ^*^p < 0.05 when vs. normal nasopharyngeal epithelial tissues. Abbreviation: NPC: nasopharyngeal carcinoma.

The expression of IL-6 in the NPC group (51.97 ± 8.02 ng/mL) was significantly higher than that in the control group (4.14 ± 0.03 ng/mL) (*p* < 0.05). The results of IHC are displayed in Figure 1A and 1B. Compared to normal nasopharyngeal epithelial tissues, the expressions and positive rates of IL-6, STAT3, p-STAT3, JAK2, p-JAK2 and CyclinD1 were obviously higher in NPC tissues (all *p* < 0.05). The stained granules in NPC tissues were closely arranged and presented remarkably brown, while the granules in normal nasopharyngeal epithelial tissues were sparsely arranged and presented yellow. Correlation of IL-6, STAT3, p-STAT3, JAK2, p-JAK2 and CyclinD1 expressions with clinicopathological features of NPC patients are presented in Table 2. The results suggested that the expressions of IL-6, STAT3, p-STAT3, JAK2, p-JAK2 and CyclinD1 were correlated with LNM and TNM stage of NPC patients (all *p* < 0.05), but had no significant difference in terms of age, gender or histological type (all *p* > 0.05). These results demonstrate that the expression of IL-6, STAT3, p-STAT3, JAK2, p-JAK2 and CyclinD1 is correlated to the development and metastasis of NPC.

**Table 2:** Correlation of IL-6, STAT3, p-STAT3, JAK2, p-JAK2 and CyclinD1 expressions with clinicopathological features of NPC patients.

**Clinicopathological features**	**Case**	**STAT3**			**p-STAT3**			**JAK2**			**p-JAK2**			**IL-6**			**CyclinD1**		
		Positive	Negative	*p*	Positive	Negative	*p*	Positive	Negative	*p*	Positive	Negative	*p*	Positive	Negative	*p*	Positive	Negative	*p*
Age				0.227			0.599			0.614			0.453			0.253			
< 45 years	52	39	13		36	16		32	20		37	15		43	9		35	17	0.518
≥ 45 years	65	42	23		42	23		37	28		42	23		48	17		40	25	
Gender				0.059			0.189			0.394			0.269			0.321			
Male	64	49	15		46	18		40	24		46	18		52	12		44	20	0,249
Female	53	32	21		32	21		29	24		33	20		39	14		31	22	
Type				0.187			0.259			0.139			0.216			0.630			
Squamous carcinoma	9	8	1		7	2		8	1		8	1		8	1		8	1	0.165
Poorly differentiated carcinoma	93	60	33		58	35		51	42		59	34		70	23		55	38	
Undifferentiated carcinoma	9	8	1		8	1		7	2		8	1		8	1		7	2	
Adenosquamous carcinoma	6	5	1		5	1		3	3		4	2		5	1		5	1	
LNM				0.032			0.048			0.001			0.001			0.003			
Yes	66	51	15		49	17		48	18		52	14		58	8		49	17	0.009
No	51	30	21		29	22		21	30		27	24		33	18		26	15	
TNM stage				0.010			0.003			0.003			0.023			< 0.001			
I + II	36	19	17		17	19		14	22		19	17		20	16		17	19	0.011
III + IV	81	62	19		61	20		55	26		60	21		71	10		58	23	

Abbreviations: IL-6: interleukin-6; STAT3: Signal transducers and activators of transcription 3; JAK2: Janus kinase 2; NPC: nasopharyngeal carcinoma; LNM: lymph node metastasis; TNM: tumor node metastasis.

### mRNA expressions of IL-6, JAK2, STAT3 and CyclinD1 are upregulated in NPC patients

**Figure 2:**
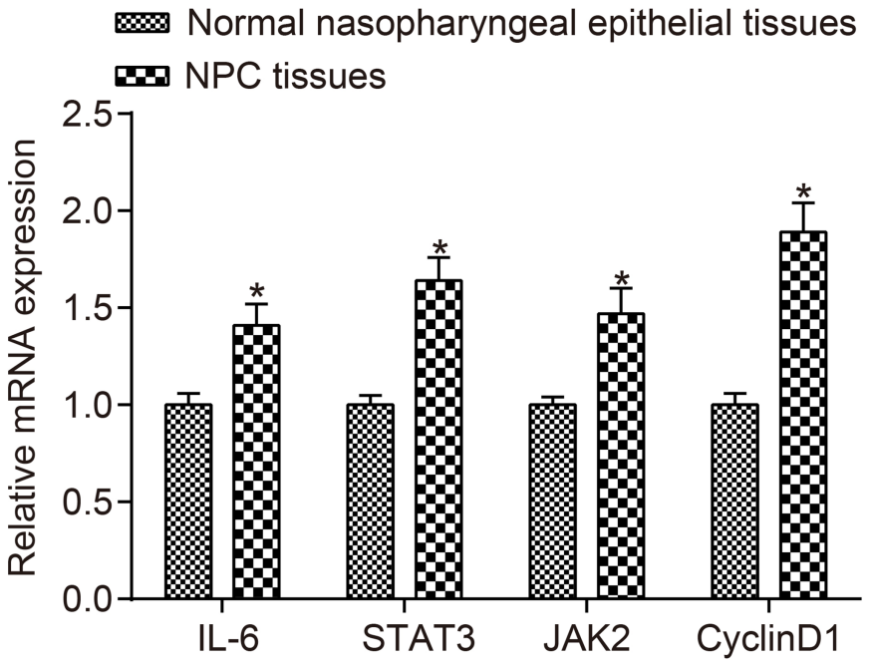
**The mRNA expressions of IL-6, JAK2, STAT3 and CyclinD1 were higher in NPC tissues than in normal nasopharyngeal epithelial tissues.** ^*^p < 0.05 when vs. normal nasopharyngeal epithelial tissues; NPC tissues (*n* = 117); Normal nasopharyngeal epithelial tissues (*n* = 112). Abbreviation: NPC: nasopharyngeal carcinoma.

The mRNA expressions of IL-6, JAK2, STAT3 and CyclinD1 in NPC tissues and normal nasopharyngeal epithelial tissues detected by RT-qPCR are displayed in Figure 2. The results revealed that compared to normal tissues, the mRNA expressions of IL-6, JAK2, STAT3 and CyclinD1 were higher in NPC tissues (all *p* < 0.05).

### Protein expressions of IL-6, STAT3, JAK2, and CyclinD1 as well as the extent of STAT3 and JAK2 phosphorylation are elevated in NPC tissues

**Figure 3:**
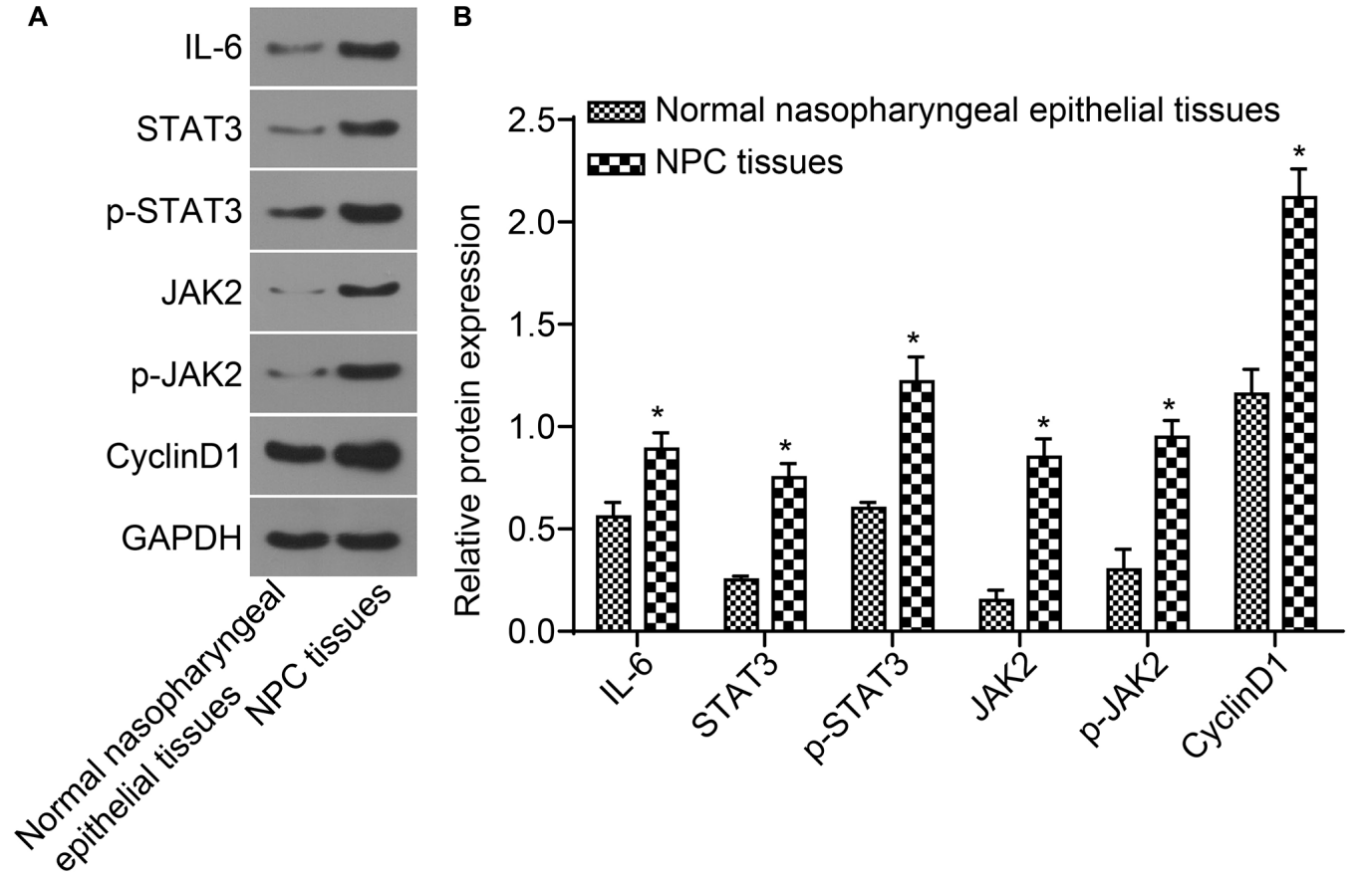
**The protein expressions of IL-6, STAT3, JAK2, and CyclinD1 as well as the extent of STAT3 and JAK2 phosphorylation in NPC tissues were higher.** (**A**) The protein band detected by western blot analysis; (**B**) Densitometry analysis of Western blot data. ^*^p < 0.05 when vs. normal nasopharyngeal epithelial tissues; NPC tissues (*n* = 117); Normal nasopharyngeal epithelial tissues (*n* = 112). Abbreviation: NPC: nasopharyngeal carcinoma.

Protein expressions of IL-6, STAT3, JAK2, and CyclinD1 as well as the level of STAT3 and JAK2 phosphorylation detected by western blot analysis are shown in Figure 3A and 3B. The protein expressions of IL-6, STAT3, JAK2, and CyclinD1 as well as the extent of STAT3 and JAK2 phosphorylation in NPC tissues were higher than those in normal nasopharyngeal epithelial tissues (all *p* < 0.05). Correlation analysis indicated that STAT3 was positively correlated with JAK2 and CyclinD1 (*r* = 0.999, *r* = 0.990, all *p* < 0.001), p-STAT3 was positively correlated with p-JAK2 and CyclinD1 (*r* = 0.994, *r* = 0.986, all *p* < 0.001). IL-6 was positively correlated with JAK2, STAT3 and CyclinD1 (*r* = 0.999, *r* = 0.999, *r* = 0.990, all *p* < 0.001).

### Elevated expression of IL-6/STAT-3 signaling pathway-related proteins is linked to reduced survival rate of NPC patients

**Figure 4:**
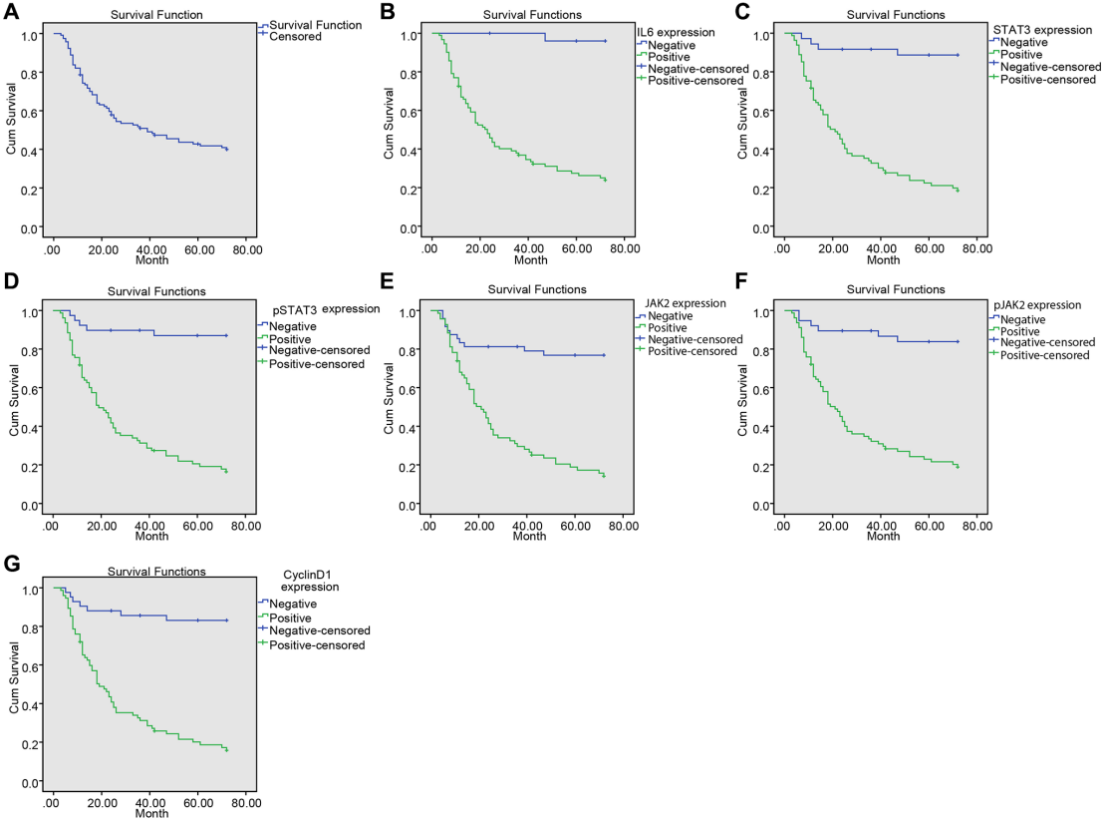
**Upregulated IL-6, SATA3, p-SATA3, JAK2, p-JAK2, CyclinD1 expression was associated with decreased survival time in NPC patients.** (**A**) Kaplan-Meier survival curve of the overall survival of NPC patients; (**B**) Kaplan-Meier survival curve of correlation between IL-6 expression and the survival time of patients; (**C**) Kaplan-Meier survival curve of correlation between STAT3 expression and the survival time of patients; (**D**) Kaplan-Meier survival curve of correlation between p-STAT3 expression and the survival time of patients; (**E**) Kaplan-Meier survival curve of correlation between JAK2 expression and the survival time of patients; (**F**) Kaplan-Meier survival curve of correlation between p-JAK2 expression and the survival time of patients; (**G**) Kaplan-Meier survival curve of correction between CyclinD1 expression and the survival time of patients.

The median survival time of 117 patients was 39 months. The 72-month survival rate was 41.03%. The effects of IL-6, STAT3, p-STAT3, JAK2, p-JAK2 and CyclinD1 expressions on the survival of NPC patients are shown in Figure 4A–4G. The result confirmed that the median survival time of patients with positive expression of IL-6, STAT3, p-STAT3, JAK2, p-JAK2 and CyclinD1 was significantly lower than those with negative expression (Table 3).

**Table 3:** Relationship between IL6/SATA-3 signaling pathway and survival time in NPC patients.

**Genes/ Proteins**	**Median survival time (month)**	***p***
IL-6		< 0.001
Positive	33	
Negative	71	
STAT3		< 0.001
Positive	31	
Negative	66	
p-STAT3		< 0.001
Positive	30	
Negative	65	
JAK2		< 0.001
Positive	30	
Negative	59	
p-JAK2		< 0.001
Positive	31	
Negative	64	
CyclinD1		< 0.001
Positive	30	
Negative	63	

Abbreviations: NP: nasopharyngeal carcinoma; IL-6: interleukin-6; JAK2: Janus kinase 2; STAT3: Signal transducers and activators of transcription 3.

### LNM and expressions of IL-6 and p-STAT3 are the risk factors for the prognosis of NPC patients

Multivariate analysis of the risk factors for prognosis is shown in Table 4. The results demonstrated that LNM and expressions of IL-6 and p-STAT3 were the risk factors for poor prognosis of NPC (all *p* < 0.05). However, gender, age, histological type and other factors had no significant effects on prognosis of NPC (all *p* > 0.05).

**Table 4:** Result of Cox proportional hazard.

	**B**	**SE**	**Wald**	***P***	**Exp (B)**	**95% CI**
Age	–0.433	0.43	1.015	0.314	0.649	0.280–1.506
Gender	0.554	0.421	1.737	0.187	1.741	0.763–3.969
Pathological stage	0.026	0.146	0.03	0.862	1.026	0.770–1.367
Pathological type	–0.067	0.232	0.083	0.773	0.935	0.593–1.474
IL-6 expression	2.419	1.172	4.257	0.039	11.231	1.129–111.757
JAK2 expression	–0.511	0.512	0.998	0.318	0.6	0.220–1.635
pJAK2 expression	0.088	0.743	0.014	0.906	1.092	0.255–4.682
STAT3 expression	0.15	1.144	0.017	0.896	1.161	0.123–10.927
pSTAT3 expression	1.972	0.842	5.484	0.019	7.185	1.379–37.436
CyclinD1expression	0.104	0.661	0.025	0.875	1.109	0.304–4.052
LNM	0.814	0.304	7.145	0.008	2.257	1.242–4.098

Abbreviations: IL6: interleukin-6; JAK2: Janus kinase 2; STAT3: Signal transducers and activators of transcription 3; LNM: Lymph node metastasis.

### IL-6 promotes cell proliferation and JAK2/STAT3 signaling pathway inhibitor attenuates cell proliferation of CNE-1 and SUNE1 cells induced by IL-6

**Figure 5:**
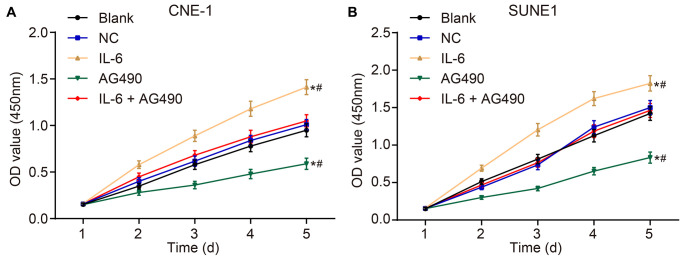
**IL-6 promotes NPC cell proliferation *in vitro*.** (**A**) Cell proliferation of CNE-1 cells *in vitro*; (**B**) Cell proliferation of SUNE1 cells *in vitro*; ^*^p < 0.05 vs. the blank group; ^#^*p* < 0.05 vs. the IL-6 + AG490 group. The experiment in each group was repeated three times. Abbreviations: NC, negative control; IL-6, interleukin-6; NPC, nasopharyngeal carcinoma.

CCK8 assay was employed to observe the cell proliferation of CNE-1 and SUNE1 cells in each group. The OD value at 450 nm was measured by an enzyme labelling instrument, which could reflect the number of living cells. The results (Figure 5A and 5B) suggested that the OD_450_ in each group had no significant difference after 1-h incubation (*p* > 0.05). Next, compared with the blank group, the OD_450_ value in the IL-6 group increased, suggesting that IL6 spurred cell proliferation (*p* < 0.05). Besides, the OD_450_ value in the AG490 group decreased, indicating that AG490 inhibited cell proliferation (*p* < 0.05). Compared with the IL-6 + AG490 group, the OD_450_ in the IL-6 group increased, which means AG490 decreased the proliferation promoted by IL6 (*p* < 0.05), and the OD_450_ in the AG490 group decreased, also referring that the proliferation was enhanced by IL6 (*p* < 0.05). Taken together, IL-6 promoted cell proliferation of CNE-1 and SUNE1 NPC cells while the inhibitor of JAK2/STAT3 signaling pathway attenuated cell proliferation induced by IL-6.

### IL-6 enhances cell migration and invasion while JAK2/STAT3 signaling pathway inhibitor attenuates migration and invasion of CNE-1 and SUNE1 cells induced by IL-6

**Figure 6:**
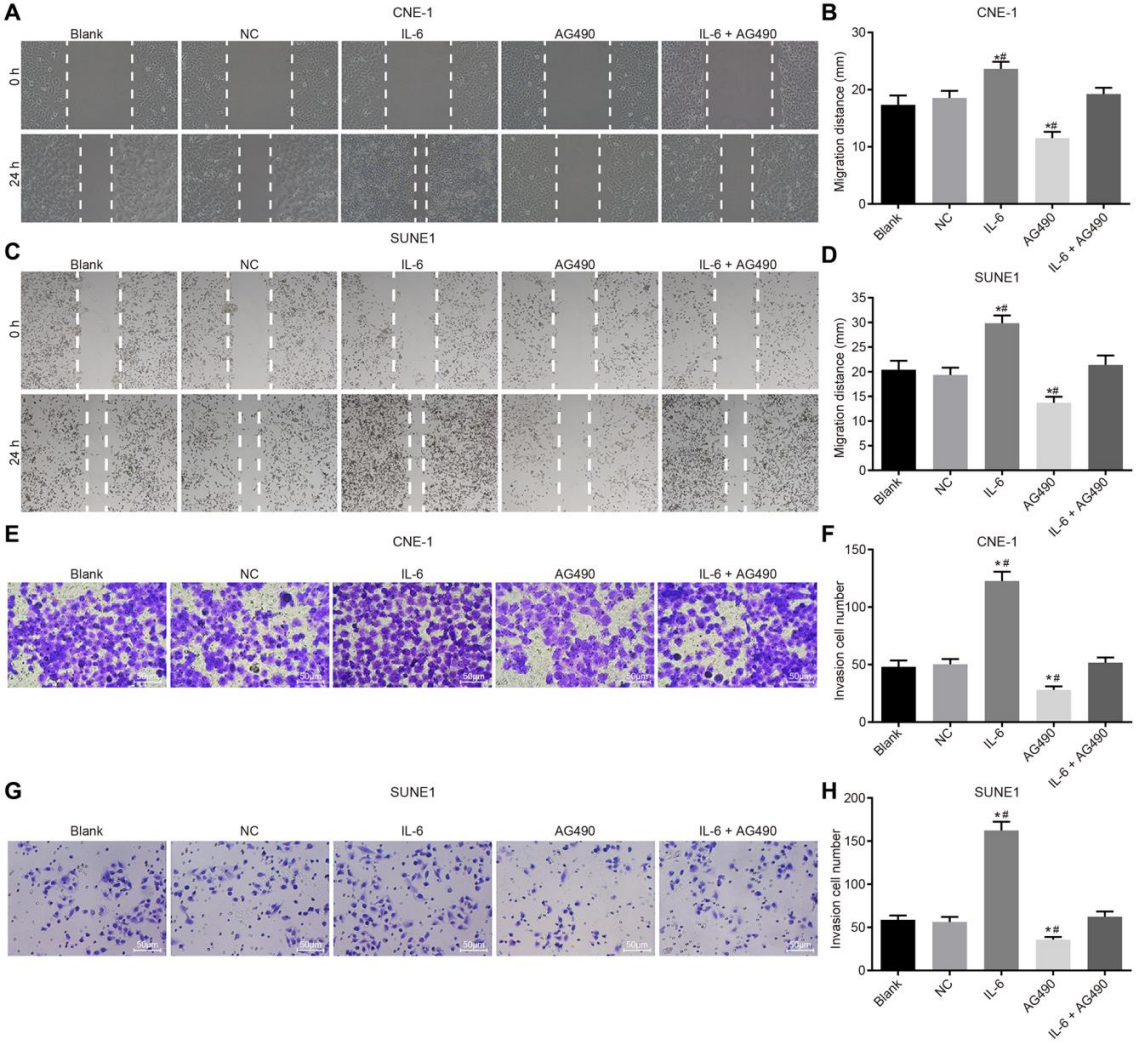
**IL-6 enhances NPC cell migration and invasion *in vitro*.** (**A**) Cell migration of CNE-1 cells (×40); (**B**) Migration distance of CNE-1 cells; (**C**) Cell invasion of SUNE-1 cells (×40); (**D**) Migration distance of SUNE1 cells; (**E**) Cell invasion of CNE-1 cells (×200); (**F**) The number of invasive CNE-1 cells; (**G**) Cell invasion of SUNE1 cells (×200); (**H**) The number of invasive SUNE1 cells. ^*^p < 0.05 vs. the blank group; ^#^*p* < 0.05 vs. the IL-6 + AG490 group. The experiment in each group was repeated three times. Abbreviations: NC: negative control; IL-6: interleukin-6; NPC: nasopharyngeal carcinoma.

Scratch test (Figure 6A–6D) and Transwell assay (Figure 6E–6H) were employed to determine cell migration and invasion of CNE-1 and SUNE1 cells. Our results shown that compared with the blank group, migration distance and the number of cells passing through Matrigel glue increased in the IL-6 group (both *p* < 0.05), while the migration distance and the number of cells passing through Matrigel glue decreased in the AG490 group (both *p* < 0.05). In addition, compared with the IL-6 + AG490 group, migration distance and the number of cells passing through Matrigel glue increased in the IL-6 group (*p* < 0.05), and migration distance and the number of cells passing through Matrigel glue in the AG490 group decreased (*p* < 0.05). And there was no obvious change in the NC group and the blank group (*p* > 0.05). According to the above results, we concluded that IL-6 promoted cell migration and invasion while JAK2/STAT3 signaling pathway inhibitor AG490 attenuates migration and invasion induced by IL-6 in CNE-1 and SUNE1 cells.

## DISCUSSION

NPC is one of the most misdiagnosed diseases and a common malignancy with a high incidence threatening patients’ life and health [40]. Although treatment can effectively control the clinical prognosis, the five-year survival rate of stage is still poor [41]. Previous study has reported that IL6 was up-regulated in gallbladder cancer tissues and promoted cell proliferation, and invasion [42]. Additionally, IL-6 and JAK2/STAT3 signaling pathway is associated with benign or proliferative diseases posing a synergistic effect on cell growth, apoptosis, tumor immunosuppression, angiogenesis, and metastasis [43, 44], so our study focuses on the expressions of IL-6 and the JAK2/STAT3 signaling pathway-related proteins in NPC and the correlation with clinicopathological features and prognosis in NPC. Collectively, we concluded that IL-6 was up-regulated in NPC and the JAK2/STAT3 signaling pathway was triggered, contributing to a poor prognosis of NPC patients. Firstly, the expressions of IL-6, JAK2, STAT3 and CyclinD1 were discovered higher in NPC tissues than in normal nasopharyngeal epithelial tissues. Accumulating evidence demonstrate that the activation of JAK2/STAT3 signaling pathway related with IL-6 plays a critical role in the growth and progression of tumors [21, 45]. Consistently, a previous study has proved that IL-6 could promote the development of NPC and the expression of IL-6 is high in NPC tissues [21]. The level of IL-6 in the blood is also a promising marker for evaluating the outcome of NPC treatment [46]. A study has confirmed that IL-6 expression contributed to the survival of head and neck cancer [47], and IL-6 expression also played a significant role in the survival of lung cancer [48]. Besides, increased expression of IL-6 has been found in a variety of tumors, such as lung cancer, gastric cancer, breast cancer and lymphoma, and is associated with poor clinical prognosis, which is consistent with our results [49]. What's more, IL-6 was confirmed to activate JAK and STAT3 by binding to IL-6 receptor and gp130–32 [49–51]. IL-6 activated the JAK2/STAT3 pathway, which accelerated cell cycle progression by promoting CyclinD1 expression in pancreatic cancer [52]. Moreover, STAT3 is able to transmit signals from the cell surface to the nucleus in the activation of growth and cytokines factors, which could improve the survival rate of tumor cells [53]. STAT3 could develop into a JAK substrate combined with SH2 (Src homology2) domain and receptor, and then p-STAT3 targets the gene transcription and mediates the expression of inflammatory cytokines so as to make benefits of cell proliferation and angiogenesis [54]. In addition, it has been illustrated that STAT3 could make a contribution to carcinogenesis and tumor progression by the way of increasing gene expression [55]. Similarly, Bollrath J has proved that STAT3/p-STAT3 is correlated with tumor progression leading to cancer, and the high expressions of STAT3 and p-STAT3 exist in many kinds of cancers [56]. A study confirmed that STAT3 is essential for proliferation and survival in colon cancer-initiating cells [57], and the IL-6/STAT3 pathway regulated the proliferation and contributed to the survival in colorectal cancer [58].

In addition, the study proved that the expressions of IL-6, STAT3, p-STAT3, JAK2, p-JAK2 and CyclinD1 were related to LNM and TNM stage of NPC. Consistently, the previous study has proved that IL-6 could promote tumor growth and metastasis, and high expression of IL-6 in breast cancer is related to the degree of lymph node involvement and TNM stage, indicating poor prognosis [59]. Activation of STAT3 by IL-6 is evidenced to participate in the growth, survival and metastasis of human tumors [60]. Additionally, Shukla et al. has found that STAT3 overexpression in cervical cancer contributed to poor prognosis of cervical cancer [61]. Besides, the expression of STAT3 in cervical cancer is higher than that in normal cervical tissues and precancerous lesions, and the expression of STAT3 in cancer tissues is related to the clinical stage, which also proves our results [62]. The phosphorylation of STAT3 is induced by JAK2, and phosphorylated STAT3 dimers are transformed into the nucleus in order to induce the expression of genes regulated by STAT, which is related to the differentiation, proliferation and survival of cells [63]. The activated or highly expressed STAT3 has been discovered in many NPC cases, which indicates that STAT3 activation results in the development and invasiveness of NPC, therefore, STAT3 could provide a promising and effective value for treatment of NPC [64]. Moreover, it has been found that the phosphorylated JAK2 is regulated by IL6 through gp130 directly, and inhibition of JAK2 could significantly reduce tumor metastasis so as to prolong the overall life expectancy of patients, suggesting that JAK2 could act as an ideal target for the prevention of tumor metastasis [64, 65]. Besides, a study has reported that IL-6 promoted cell migration and invasion in NPC [66], and Stat3 inhibition suppressed IL-6 induced cell proliferation in NPC [21]. Moreover, IL-6 also increased cell proliferation, migration and invasion by activating JAK2/STAT3 signaling pathway in human glioma cells [67]. In our study, we found enhanced cell proliferation, migration and invasion when IL-6 was activated in NPC. So, we considered that IL-6 overexpression may promote cell proliferation, migration and invasion in NPC.

In conclusion, activation of JAK2/STAT3 signaling pathway and up-regulation of IL-6 is correlated with the survival rate of NPC patients. LNM and the overexpression of IL-6 and STAT3 are risk factors for poor prognosis of NPC. Therefore, IL-6 overexpression and activation of JAK2/STAT3 pathway could become a new therapeutic target for NPC treatment. However, the study is still in the preclinical stage, and the investigation of the mechanism of action is insufficient. Further experiments are required in order to further clarify the intrinsic mechanisms.
